# Pdcd4 deficiency enhances macrophage lipoautophagy and attenuates foam cell formation and atherosclerosis in mice

**DOI:** 10.1038/cddis.2015.416

**Published:** 2016-01-21

**Authors:** L Wang, Y Jiang, X Song, C Guo, F Zhu, X Wang, Q Wang, Y Shi, J Wang, F Gao, W Zhao, Y H Chen, L Zhang

**Affiliations:** 1 Department of Immunology, Shandong University School of Medicine, Jinan, China; 2 Department of Hematology, the Second Hospital of Shandong University, Jinan, China; 3 Basic Research Center, Shandong Cancer Hospital, Jinan, China; 4 Key Laboratory of Cardiovascular Remodeling and Function Research, Qilu Hospital, Shandong University, Jinan, China; 5 Department of Pathology and Laboratory Medicine, University of Pennsylvania School of Medicine, Philadelphia, PA, USA

## Abstract

Macrophage foam cells, a major component of the atherosclerotic lesion, have vital roles in the development of atherosclerosis. Lipoautophagy, a type of autophagy characterized by selective delivery of lipid droplet for lysosomal degradation, may impact atherosclerosis by regulating macrophage foam cell formation. Previously, we reported that programmed cell death 4 (PDCD4), a tumor suppressor, negatively regulated autophagy in tumor cells. However, its roles in macrophage lipoautophagy, foam cell formation and atherosclerosis remain to be established. Here we found that Pdcd4 deficiency clearly improved oxidized low-density lipoproteins-impaired autophagy efflux, promoted autophagy-mediated lipid breakdown in murine macrophages and thus prevented macrophage conversion into foam cells. Importantly, Pdcd4 deficiency in mice significantly upregulated macrophage autophagy in local plaques along with attenuated lipid accumulation and atherosclerotic lesions in high-fat-fed Apolipoprotein E knockout mice. Bone marrow transplantation experiment demonstrated that PDCD4-mediated autophagy in hematopoietic cells contributed to the development of atherosclerosis. These results indicate that endogenous PDCD4 promotes for macrophage foam cell formation and atherosclerosis development via inhibiting autophagy and provides new insights into atherogenesis, suggesting that promoting macrophage autophagy through downregulating PDCD4 expression may be beneficial for treating atherosclerosis.

Atherosclerosis is a lipid dysfunction-derived chronic inflammatory process in large and medium arterial wall.^1^ Macrophage foam cell, as a major component in the lesion of atherosclerosis, has vital role in the development of atherosclerosis. In the initial step of atherosclerotic development, circulating monocytes migrate into arterial wall via dysfunctional endothelial cells and differentiate into macrophages.^2, 3, 4^ The infiltrated macrophages ingest and digest oxidized low-density lipoprotein (ox-LDL), and then transport lipid out of vascular wall.^5^ However, macrophage with overloaded lipids stored in the form of lipid droplets (LDs) will transform into foam cells. Macrophage foam cell formation could promote the development of atherosclerosis.^6^ Thus, decreasing the formation of macrophage foam cell would be an attractive strategy to reverse plaque lipid buildup.^7^

The macroautophagy (hereafter referred to as autophagy) is an evolutionarily conserved and well-controlled cellular catabolic process. During the process, cytoplasmic components are sequestered in double-membrane vesicles (which is called autophagosome) and degraded by fusion with lysosomal compartments (autophagolysosome) for recycling application.^8^ The process of autophagy is regulated by several autophagy-related genes (ATGs) encoded proteins, such as ATG5, ATG6 (also known as BECN1), ATG8 (also known as microtubule-associated protein 1 light chain 3, LC3) and ATG12. ATG5 is involved in the early stage of autophagosome formation. ATG5 is conjugated with ATG12 and ATG16L to form ATG12–ATG5–ATG16L complex, which contributes to the elongation and closure of the autophagosomes in the generation of lipidated forms of LC3 family proteins.^9^ Lipoautophagy, a type of autophagy that selectively delivers LDs for lysosomal degradation,^10^ regulates lipid metabolism and is involved in the process of atherosclerosis.^11, 12, 13, 14^ In advanced atherosclerosis, macrophage autophagy becomes dysfunctional. However, the basic autophagy deficiency in macrophage by specific Atg5 knockout accelerates atherosclerotic plaques in high-fat-fed *ldlr*^*−/−*^ mice via promoting oxidative stress, plaque necrosis^12^ or inflammasome hyperactivation.^13^ More interestingly, autophagy can enhance brokendown of lipid in LD, cholesterol efflux from macrophage foam cells and further inhibit atherogenisis.^14^ Stent-based delivery of everolimus (mTOR inhibitor) in atherosclerotic plaques of cholesterol-fed rabbits leads to a marked reduction of macrophages via autophagic cell death.^15^ Therefore, regulating the level of macrophage autophagy and macrophage conversion into foam cells would be a potential target for preventing the atherosclerotic plaques formation.^16^

Programmed cell death 4 (PDCD4), an inhibitor of protein translation, inhibits translation initiation via binding to the translation initiation factor eIF4A or translation elongation by direct or indirectly binding to the coding region of specific RNAs.^17, 18^ Accumulated evidence has demonstrated PDCD4 as a tumor suppressor.^19^ PDCD4 can inhibit promotion and progression of tumors, such as lung cancer,^20^ hepatocellular carcinoma cells,^21^ colon cancer,^22^ ovarian cancer^23^ and glioma.^24^ In addition, it has been reported that PDCD4 is also involved in the development of inflammatory diseases.^25, 26, 27, 28, 29, 30^ For example, Pdcd4-deficient mice are resistant to experimental allergic encephalitis,^25^ LPS-induced endotoxin shock^26^ and type-1 diabetes.^27^ In addition, Pdcd4-deficient mice are sensitive to LPS/D-galactosamine-induced acute liver injury.^28^ Recently, we reported that Pdcd4 deficiency attenuated adipocyte foam cells, diet-induced obesity, obesity-associated inflammation and insulin resistance,^29^ and increased IL-10 expression by macrophages that partly involved in atherosclerosis in hyperlipidemic mice,^30^ suggesting that PDCD4 may be involved in the metabolism-related diseases. Furthermore, we found that PDCD4 negatively regulated autophagy by inhibiting ATG5 expression in tumor cells.^31^ However, its role in macrophage lipoautophagy and foam formation, and association with atherosclerosis remain to be investigated.

In the present study, we found that Pdcd4 deficiency improved ox-LDL-impaired autophagy efflux in murine macrophage and subsequently attenuated macrophage conversion into foam cells in an autophagy-dependent manner and further attenuated the formation of atherosclerotic lesions in hyperlipidemia mice. These results indicate that PDCD4 is critical for macrophage foam cell formation in atherosclerosis development and provides new insights into atherogenesis, and potential therapeutic avenues to treat atherosclerosis-associated diseases.

## Results

### Pdcd4 deficiency improved ox-LDL-impaired autophagy efflux in murine macrophages

**Figure 1:**
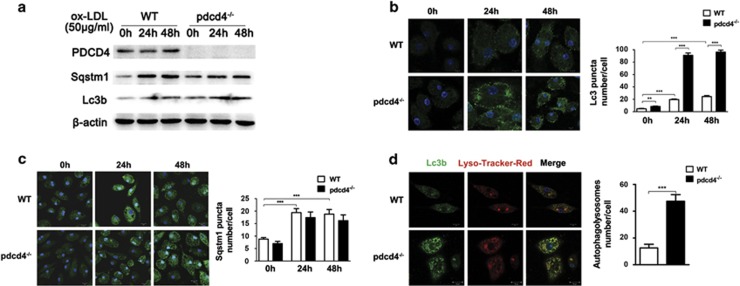
Pdcd4 deficiency improved ox-LDL-impaired autophagy efflux in murine macrophages. Primary peritoneal macrophages from WT or pdcd4^−/−^ mice were stimulated by 50 *μ*g/ml ox-LDL for 24 h or 48 h. Levels of Lc3b, Sqstm1 and PDCD4 expression were detected by western blot (**a**). Immunofluorescence was used to detect Lc3b^+^ autophagosomes (**b**) and SQSTM1 expression (**c**). Autophagolysosomes were detected by LysoTracker Red (red) and anti-Lc3b conjugated with Alexa 488 (green, **d**). Data were representative of three independent experiments. **P<0.01; ***P<0.001

Increasing evidence demonstrates that macrophage autophagy has important roles in atherosclerosis.^32^ To investigate the influence of PDCD4 on macrophage autophagy induced by toxic lipid, peritoneal macrophages from wild-type (WT) and Pdcd4 knockout (pdcd4^−/−^) mice were stimulated with atherosclerosis-related stimulator, ox-LDL, for 24 and 48 h, and then autophagy level was detected by western blot and immunofluorescence staining. As shown in Figure 1, ox-LDL stimulation for 24 or 48 h greatly increased the expression of Lc3b by western blot (Figure 1a) and the number of Lc3b^+^ (major marker of autophagy) autophagosomes by immunofluorescence staining (Figure 1b), and meanwhile enhanced the level of Sqstm1/p62 (Figures 1a and c), a critical chaperone for removing protein aggregates via autophagolysosome,^33^ in macrophages from WT mice. As the accumulation of Sqstm1 protein is a feature of autophagic efflux interruption in toxic lipid-induced autophagy,^10^ these results suggested that ox-LDL causes dysfunction of autophagy efflux. However, Pdcd4 deficiency markedly increased the expression of Lc3b and the number of Lc3b^+^ autophagosomes induced by ox-LDL, but decreases the accumulation of Sqstm1 protein (Figures 1a–c), suggesting that Pdcd4 deficiency may improve ox-LDL-impaired autophagy efflux.

Autophagosomes fuse with lysosome to form autophagolysosome at the end of autophagy efflux, and then facilitate the degradation and recycling of cytoplasmic contents delivered by autophagosome. Thus, the number of autophagolysosome and function of lysosome reflect the status of autophagy efflux. To further address the potential role of PDCD4 in autophagy efflux, we detected the influence of PDCD4 on autophagolysosome formation by double-fluorescent staining. Autophagosome was labeled by anti-Lc3b antibody and functional lysosome was stained by LysoTracker Red, a lysosomotropic dye that shows red color in acidic pH lysosome. As shown in Figure 1c, Pdcd4 deficiency greatly increased the number of autophagolysosome. Collectively, these data indicate that Pdcd4 deficiency improved ox-LDL-damaged autophagy efflux in macrophages.

### Pdcd4 deficiency enhanced lipoautophagy-mediated breakdown of lipid in LD

**Figure 2:**
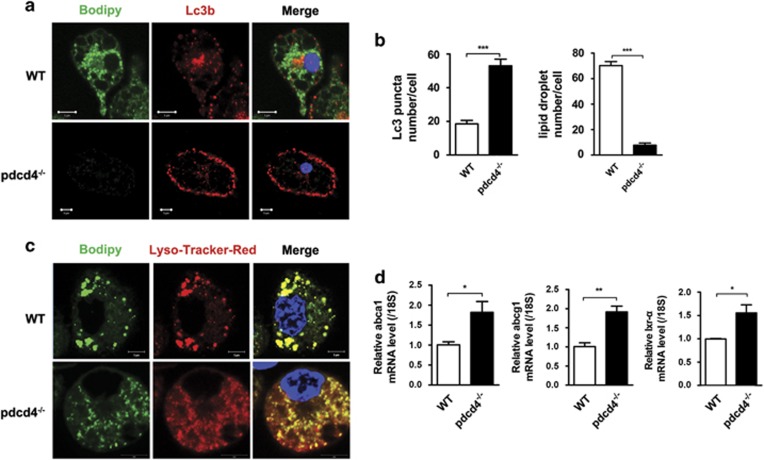
Pdcd4 deficiency enhanced autophagy-mediated breakdown of lipid in LD. Primary peritoneal macrophages from WT or pdcd4^−/−^ mice were stimulated by 50 *μ*g/ml ox-LDL for 24 h. LD was stained with Bodipy to demonstrate neutral lipid (green) and anti-Lc3 conjugated with Alexa 555 to show autophagosome (red, **a**). The numbers of Lc3b^+^ autophagy puncta and Bodipy^+^ LDs per cell were accounted (**b**) and LD stained by Bodipy (green) was colocalized by lysosome, stained by LysoTracker Red (red, **c**). Real-time PCR was applied to show Abca1, Abcg1 and Lxr-*α* expression in macrophages (**d**). Data were representative of three independent experiments. *P<0.05; **P<0.01; ***P<0.001

The LD is the major site of cholesterol storage in macrophage foam cell. It can be selectively delivered into lysosome for degradation via autophagy.^7, 11^ To investigate whether the Pdcd4 deficiency improved autophagy efflux also impact the breakdown of lipid in LD, autophagosome was labeled by anti-Lc3b antibody, LD was stained by Bodipy, which is commonly used to fluorescently stain neutral lipids, and lysosome was stained by LysoTracker Red. In consistent with previous finding in which autophagy-mediated breakdown of lipid in LD in macrophage,^11^ we found that cells with more Lc3b^+^ autophagosomes showed less Bodipy^+^ LDs, whereas cells with less Lc3b^+^ autophagosomes revealed more Bodipy^+^ LDs, indicating autophagy-mediated lipid breakdown in LD (Supplementary Figure S1). Furthermore, Pdcd4 deficiency led to an increase in the number of Lc3b^+^ autophagosomes along with a decrease in the number of Bodipy^+^ LDs (Figures 2a and b). More importantly, Bodipy^+^ LDs colocalizated with fewer and larger lysosomes, a morphological feature for lysosomal storage disorders,^34, 35^ in WT macrophages but do with more and smaller lysosomes, a feature of new reformed functional lysosome,^36^ in Pdcd4 knockout mice (Figure 2c), suggesting that Pdcd4 deficiency promotes lipid breakdown in LD via elevating autophagy and improving lysosome function.

Since lipid efflux is closely linked to the ATP-binding cassette (ABC) transporter, including ABCA1 and ABCG1 that are regulated upstream transcriptional factor liver x receptor-*α* (LxR-*α*).^37, 38^ Our previous research has showed that Pdcd4 negatively regulates expression of Lxr-*α* in adipocyte.^29^ So, we further investigated the impact of Pdcd4 on the expression of Abca1, Abcg1 and Lxr-*α* by real-time PCR. As shown in Figure 2d, Pdcd4 deficiency enhanced Abca1, Abcg1 and Lxr-*α* expression, which was helpful for lipid efflux.

### Pdcd4 deficiency suppressed macrophage conversion into foam cells in an autophagy-dependent manner

**Figure 3:**
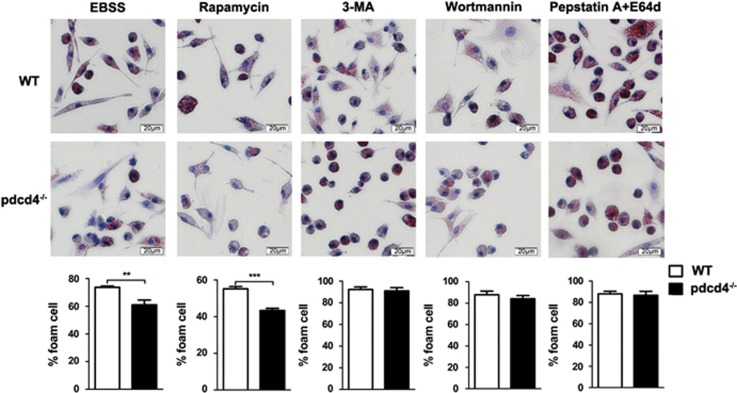
Pdcd4 deficiency suppressed macrophage conversion into foam cells in an autophagy-dependent manner. Cells were stimulated by 50 *μ*g/ml ox-LDL in starvation condition (EBSS) or in the presence of rapamycin, 3-MA, wortmannin and pepstatin A plus E64d for 24 h, and then cells were stained by oil red O. Data came from three independent experiments. **P<0.01; ***P<0.001

Macrophage with overloaded lipids stored in the form of LDs will transform into foam cells. Next, we analyzed the role of PDCD4 in macrophage foam cell formation and established the relation between autophagy and foam cell formation. Two promoting autophagy factor or agents, starvation (EBSS) and rapamycin, were used to induce autophagy. Two autophagy inhibitors, 3-methyladenine (3-MA) and wortmannin, and autophagic flux blockers, pepstatin A plus E64d, were used to inhibit autophagy, and then the macrophage foaming was analyzed. As shown in Figure 3, Pdcd4 deficiency caused reduction of macrophage foam cells in starvation or rapamycin-treated macrophages. However, Pdcd4 deficiency-alleviated foam cell formation was restored by 3-MA, wortmannin and pepstatin A plus E64d treatment. Taken together, our data indicate that Pdcd4 deficiency suppresses macrophage conversion into foam cells in an autophagy-dependent manner.

### Pdcd4 deficiency significantly upregulated macrophage autophagy in atherosclerotic plaques along with attenuated atherosclerotic lesions in high-fat-fed apoe^−/−^ mice

**Figure 4:**
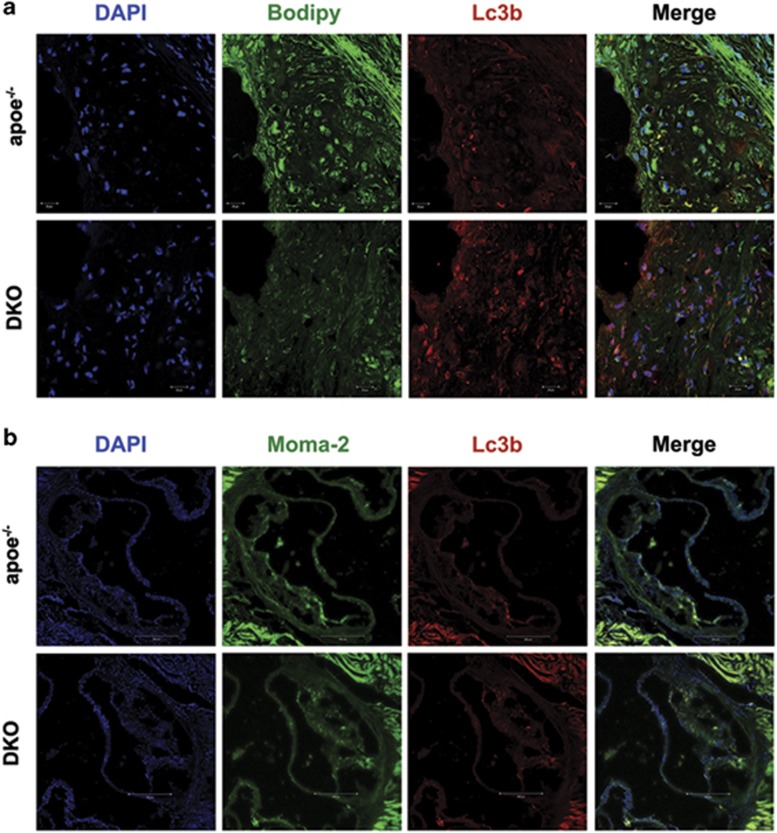
Pdcd4 deficiency promoted macrophage autophagy and attenuated lipid accumulation in plaques. apoe^−/−^ and pdcd4^−/−^apoe^−/−^ (DKO) mice were fed with a high-fat diet from 8 to 16 weeks to induce atherosclerotic lesions (apoe^−/−^: *n*=7; DKO: *n*=5). Autophagosome and LD were colocalized by double staining with anti-Lc3b antibody conjugated with Alexa 555 (red) and Bodipy (green) in aortic root (red, **a**). Macrophage and autophagosome were colocalizated by staining with anti-Moma-2 antibody conjugated with Alexa 488 (green) and anti-Lc3b antibody conjugated with Alexa 555 (red, **b**)

Macrophage autophagy has an important role in the development of atherosclerosis. To investigate the role of PDCD4-mediated macrophage autophagy in atherosclerosis, we generated Pdcd4 and Apolipoprotein E (Apoe) double-knockout (DKO) mice by crossing pdcd4^−/−^ with apoe^−/−^ mice. The mice were fed with a high-fat diet from 8 to 16 weeks to induce early atherosclerotic lesions or to 24 weeks to induce advanced atherosclerotic lesions. The size of atherosclerotic lesion and autophagy levels was analyzed as described in methods. As shown in Figure 4a, the number of autophagosomes was increased and the content of neutral lipid was decreased in local of atherosclerotic lesion of DKO mice compared with those in apoe^−/−^ mice. Furthermore, the increased LC3 puncta by Pdcd4 deficiency almost localized in macrophages (Figure 4b), which is consistent with previous report that autophagic markers mainly co-localize in monocyte–macrophages in the atherosclerotic plaques of apoe^−/−^ mice.^12^ Our data suggest that Pdcd4 deficiency enhanced autophagy in macrophage-rich region of atherosclerotic lesion.

**Figure 5:**
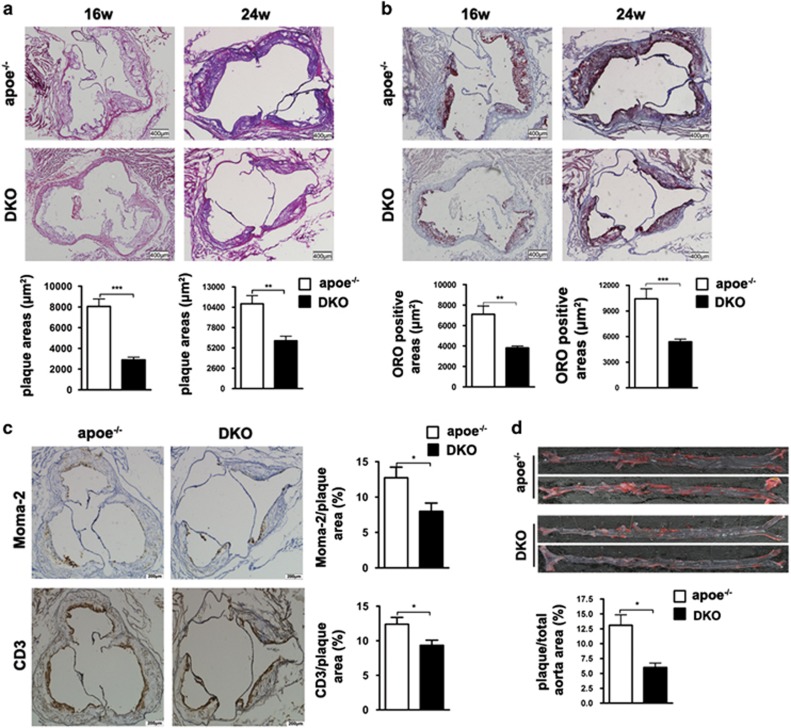
Pdcd4 deficiency attenuated the formation of atherosclerotic lesions in hyperlipidemia mice. apoe^−/−^ and pdcd4^−/−^apoe^−/−^ (DKO) were fed with a high-fat diet from 8 to 16 weeks (apoe^−/−^: *n*=7; DKO: *n*=5) or to 24 weeks to induce atherosclerotic lesions (apoe^−/−^: *n*=5; DKO: *n*=7). H&E staining was performed to show atherosclerotic lesions (**a**) and oil red O staining was applied to show lipid in aortic root (**b**). Sections of aortic root were stained for macrophages (Moma-2) and T cells (CD3; original magnification × 200) in relation to total wall area (**c**). Face lesions on thoracic–abdominal aorta were stained with oil red O (**d**). *P<0.05; **P<0.01; ***P<0.001

Along with increased autophagy level in local of atherosclerotic lesion of DKO mice, Pdcd4 deficiency caused a >50% decrease in the lesion formation in aortic root compared with that of apoe^−/−^ mice at both two time points (16 and 24 week; Figure 5a). Accordingly, Pdcd4 deficiency reduced the contents of lipid by oil red O (ORO) staining in plaques (Figure 5b) and the number of infiltrated immune cells, Moma-2^+^ macrophages and CD3^+^ T cells in the plaques (Figure 5c). More importantly, DKO mice developed less extensive face lesions in the aortic wall compared with apoe^−/−^ mice at 24 weeks (Figure 5d). However, no difference was observed between two groups of mice with regard to survival, morbidity, behavior during the experimental periods and body weight, blood glucose level or serum lipid profile, including plasma total cholesterol (TC), triglycerides (TGs), LDL and high-density lipoprotein (HDL) levels (Supplementary Table S2) at end of experiment.

Collectively, these data indicated that Pdcd4 deficiency in mice upregulated significantly macrophage autophagy in local plaque along with attenuated atherosclerotic lesions in high-fat-fed apoe^−/−^ mice.

### PDCD4-mediated autophagy in hematopoietic compartment contributed to the development of atherosclerosis

**Figure 6:**
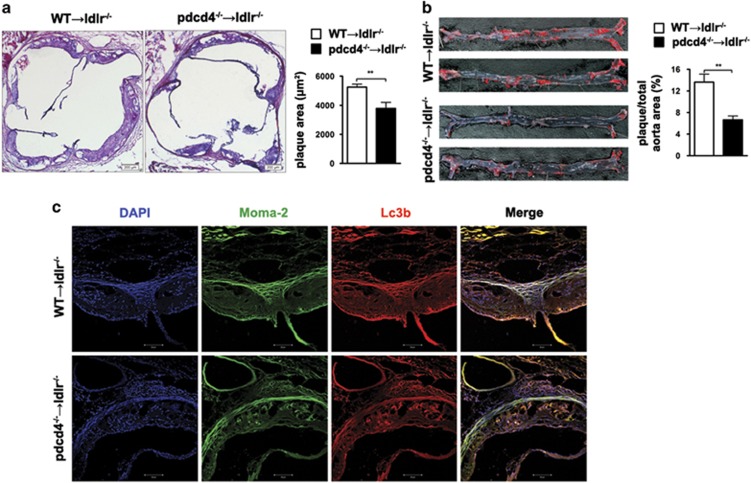
Pdcd4-mediated autophagy in bone marrow-derived cells contributed to the development of atherosclerosis. Eight-week-old female Ldl receptor-deficient (ldlr^−/−^) mice were irradiated and transplanted bone marrow cells from WT or pdcd4^−/−^ mice (*n*=7 for each group). After bone marrow transplantation, mice were maintained under a pathogen-free condition and given a high-fat diet for 16 weeks to induce atherosclerosis. H&E staining was performed to show atherosclerotic lesions (**a**). Oil red O staining was applied to show aortic face lesions (**b**). Aortic sections were stained with anti-Moma-2 conjugated with Alexa 488 to demonstrate macrophage, and with anti-Lc3b conjugated with Alexa 555 to show autophagosome (**c**). **P<0.0

To establish relation of PDCD4-mediated macrophage autophagy in the development of atherosclerosis *in vivo*, we transfused bone marrow cells derived from WT or pdcd4^−/−^ mice into irradiated ldlr^−/−^ (WT→ldlr^−/−^ and pdcd4^−/−^→ldlr^−/−^) mice, respectively. After 16 weeks of post-transplantation high-fat diet feeding, blood cell genotypes were detected to determine whether bone marrow transplantation was successful (Supplementary Figure S2). Coincidentally, the aortic atherosclerotic lesions were reduced significantly in pdcd4^−/−^→ldlr^−/−^ mice, compared with that in WT→ldlr^−/−^ mice (Figure 6a). The aortic face lesions by ORO staining were less in pdcd4^−/−^→ldlr^−/−^ mice than that in WT→ldlr^−/−^ mice (Figure 6b).

Meanwhile, the number of autophagosomes labeled by Lc3b was increased (Figure 6c) in lesion of pdcd4^−/−^→ldlr^−/−^ group mice compared with that in WT→ldlr^−/−^ group. The autophagosomes were localized in macrophages labeled by Moma-2. These results implied that PDCD4-mediated autophagy in macrophages, but not that in SMCs and ECs, contributed to the attenuation of atherosclerosis plaque formation. In addition, no significant differences of body weight and lipid levels were observed between two groups (Supplementary Table S3). Taken together, these data indicated deficiency of Pdcd4 in hematogenous cells contributed to the attenuation of atherosclerosis plaque formation.

## Discussion

Our previous work has demonstrated that PDCD4 suppresses autophagy in tumor cells via inhibiting ATG5.^31^ Here we demonstrate that PDCD4 inhibits macrophage autophagy induced by ox-LDL, which expands the inhibitory effects of PDCD4 on autophagy. Importantly, Pdcd4 deficiency enhanced macrophage lipoautophagy and attenuated macrophage foam cell formation and atherosclerosis. Our data suggest that promoting macrophage autophagy through downregulating PDCD4 expression may be beneficial in atherosclerosis-related diseases.

Recent studies have implicated that lipoautophagy, a special kind of autophagy induced by toxic lipids, contributes to the transfer of cholesterol egress from lipid-laden cells to HDL via lysosomome.^11, 35^ Autophagy can have a role in the hydrolysis of stored cholesterol droplets in macrophages, thus facilitating cholesterol efflux. In the present study, several lines of evidence indicate that PDCD4 controls autophagy-dependent lipid brokendown. First, co-localization analysis of autophagosomes and LDs demonstrates that Pdcd4 deficiency leads to an increase in the number of autophagosomes and a decrease in the number of LDs, both of which appears a negative relationship, indicating Pdcd4 deficiency increases macrophage autophagy, which digests cholesterol ester to flow out from macrophage. Second, it has been reported that cholesterol ester is hydrolyzed in lysosomes.^11^ Here Pdcd4 deficiency results to an increase in the number of activated lysosomes, co-localizing with autophagosomes and LDs. Therefore, lysosome activities contribute to the regulation of PDCD4 on foam cell formation. Third, blockage of autophagic flux by pepstatin A plus E64d aggravates macrophage conversion into foam cells. Autophagic flux is the process that autophagosome delivers cell contents to lysosome and fuses with it for digestion,^11, 35^ suggesting that PDCD4-mediated autophagy is involved in the process of lipid hypolysis and efflux. Pdcd4 deficiency promotes lysosome activities, and this may be caused by increased autophagy after Pdcd4 knockout, or acts as the result of PDCD4 direct regulation, which needs more investigation. Fourth, ABC transporter, ABCA1 and ABCG1, is closely linked to cholesterol efflux.^37, 38^ Our data showed that Pdcd4 deficiency upregulates the expression of Abca1, Abcg1 and their upstream regulator Lxr-*α*. As a protein transcription and translation suppressor, PDCD4 has been reported to regulate multiple genes expression. Our previous research has revealed that PDCD4 could bind with Lxr-*α* mRNA,^29^ thus suppresses its expression. This may be one of the regulating pathways of PDCD4 on ABCA1 and ABCG1. Taken together, PDCD4 controls autophagy-mediated cholesterol efflux.

Atherosclerosis is a chronic inflammatory process within the wall of arteries, which is responsible for most of the morbidity and mortality of cardiovascular diseases all over the world. The atherosclerotic plaque is characterized by an accumulation of lipids under the intima of artery wall, together with infiltration of immunocytes, such as macrophages, which are believed to participate in the process of atherogenesis.^2, 4^ Here we demonstrate that deficiency of Pdcd4, especially hematogenous Pdcd4, contributed to the attenuation of atherosclerosis plaque formation in hyperlipidemia mice. Autophagy in atherosclerosis has been extensively investigated with particular focus on vascular SMCs and ECs,^39, 40, 41^ and in current study bone marrow transplantation is applied to eliminate the effect of autophagy occurring in SMCs and ECs, demonstrating that autophagy in macrophage but not that in SMCs and ECs contributes to the Pdcd4 deficiency-induced attenuation of atherosclerosis.

Taken together, our analysis of Pdcd4-deficient mice and foam cell formation demonstrated the important role of PDCD4 on regulation of autophagy-dependent cholesterol efflux, foam cell formation and atherosclerosis. Thus, our findings suggest interesting prospects for therapeutic interventions.

## Materials and Methods

### Mice

apoe^−/−^ mice based on C57BL/6 background and C57BL/6 mice were purchased from Beijing University (Beijing, China). Pdcd4 knockout (Pdcd4^−/−^) mice on C57BL/6 background were generated as described previously^25^ and were mated with apoe^−/−^ mice to obtain pdcd4^−/−^apoe^−/−^ (DKO) mice. Mice used in this study were 6–8 weeks old, weighed 20–22 g and housed in the Shandong University Medical School Animal Care Facility under pathogen-free conditions, according to institutional guidelines. All animal study protocols were approved by the Animal Care and Utilization Committee of Shandong University.

### Foam cell formation and analysis

Peritoneal macrophages from Pdcd4^−/−^ and sex-matched WT mice at 6–8 weeks old were cultivated in six-well flat bottom plates with DMEM (Gibco, Invitrogen, Grand Island, NY, USA) supplemented with 10% FBS and incubated in humidified 5% CO_2_ at 37 °C for 2 h to allow macrophage adherence. The non-adherent cells were removed by three washes with DMEM. The next day, macrophages were stimulated with 50 *μ*g/ml of ox-LDL (Beijing Union Biology Co Ltd, Beijing, China) for 24 and 48 h. Cells were subjected to EBSS (Sigma-Aldrich Shanghai Trading Co Ltd, Shanghai, China) or incubated with rapamycin to induce autophagy, and incubated with 3-MA (Sigma-Aldrich Shanghai Trading Co Ltd), wortmannin (Sigma-Aldrich Shanghai Trading Co Ltd) or pepstatin A (Sigma-Aldrich Shanghai Trading Co Ltd) plus E64d (Sigma-Aldrich Shanghai Trading Co Ltd) to inhibit autophagy. Cells were then stained with ORO and rinsed in 60% isopropanol and water. Multiple images were then taken, and the percentage of stained cells was quantified. The three random independent locations were used for one experiment repeated over three independent experiments for the final calculation and comparison.

### Induction of atherosclerosis

Sex-matched apoe^−/−^ and pdcd4^−/−^apoe^−/−^ (DKO) mice received a high-fat diet (0.25 cholesterol and 15% cocoa butter) for 8 weeks (apoe^−/−^: *n*=7; DKO: *n*=5) to induce early atherosclerotic plaques or 16 weeks (apoe^−/−^: *n*=5; DKO: *n*=7) to induce advanced atherosclerotic plaques starting from 8 weeks of age.

### Bone marrow transplantation

Eight-week-old female Ldl receptor-deficient (ldlr^−/−^) mice were irradiated (using Philips 250kV orthovoltage unit, 4 rad/min) with 11 Gray, a lethal dose split into two half doses and delivered 4 h apart as recipient. Six hours later, each mouse transplanted 5–10 × 10^6^ BM cells from donor WT or pdcd4^−/−^ mice (8–12 weeks old) via tail vein injection. To prevent infection, 1 week before and 2 weeks post the bone marrow transplantation, 100 mg/l neomycin and 10 mg/l polymyxin B sulfate (Sigma-Aldrich Shanghai Trading Co Ltd) were added to the acidified drinking water. Mice were maintained under a pathogen-free condition and given a high-fat diet for 16 weeks (*n*=7 for each group).

### Histopathology and immunohistochemistry

After mice were killed, the heart with attached aortic root were removed and fixed in 4% paraformaldehyde overnight, and then embedded in OCT compound at −20 °C. Serial frozen sections (6-*μ*m thick) were cut along the aortic root up to 200 *μ*m until the valve leaflets were no longer detectable. The sections of the aorta root with aortic valve were stained with H&E and ORO for appearance and lipid. The different histological stains were observed using an Olympus microscope (IX71; Olympus Corporation, Tokyo, Japan). The ascending and descending thoracic–abdominal aorta was stained with ORO and digitally photographed at a fixed magnification. The area of lesion and positive staining was measured using Image Proplus 6.0 software (Media Cybernetics, Inc., Rockville, MD, USA) and the ratio between them was calculated. Immunohistochemistry was carried out as previously described. Corresponding sections on separate slides were stained for macrophages using a rat anti-mouse monocyte–macrophage-specific antibody (Moma-2; AbD Serotec, Oxford, UK), for CD3^+^ T cells by rat anti-mouse CD3 antibody (KT3; AbD Serotec). The slides were blocked for endogenous peroxidase activity, preincubated with goat serum, and then stained with primary antibodies by incubating for 1 h at room temperature. Secondary staining with HRP-conjugated or goat anti-rat IgG was carried out using a MaxVision Kit and a DAB Peroxidase Substrate Kit (Maixin.Bio, Fuzhou, China). Negative controls for the specificity of immunohistochemical reactions were carried out by replacing the specific primary antibody with IgG of non-immunized rat (Beyotime, Beijing, China). Immunohistochemistry was carried out twice for each sample. The different histological stains were observed using an Olympus microscope (IX71; Olympus Corporation). The area of plaque and positive staining was measured using Image Proplus 6.0 software.

### Western blots

Proteins obtained from cells were isolated and 20 *μ*g of protein were separated on SDS-polyacrylamide gel. Proteins were transferred to PVDF membranes (Millipore, Billerica, MA, USA) after electrophoresis. Membranes were then blocked with 5% bovine serum albumin in TBST containing 0.1% Tween-20 for 2 h and were probed overnight at 4 °C with the following primary antibodies: rabbit monoclonal antibodies against mice PDCD4, Lc3, *β*-actin and Sqstm1 (all 1 : 1000; CST (Shanghai) Biological Reagents Company Limited), followed by secondary antibody conjugated with HRP (Jackson Immuno Research, West Grove, PA, USA) for 1 h at room temperature. After washing, membranes were visualized by ECL detection system (Millipore).

### Immunofluorescence

Cells were cultured on Glass Bottom Cell Culture Dishes (NEST Biotechnology Co. Ltd, 801002, Wuxi, China) for detection by laser scanning confocal microscope (1.0 × 10^5^ cells per 35-mm dish), and then subjected to treatments as indicated. Cells were fixed with 4% paraformaldehyde and permeabilized with 0.1% Triton X-100 for 15 min. After incubation for 1 h with anti-Lc3 or anti-Sqstm1 (1 : 300, CST (Shanghai) Biological Reagents Company Limited), cells were washed with PBS and then incubated for 1 h with Alexa 488-conjugated (1 : 1000; Beyotime) or Alexa 555-conjugated (1 : 500; Beyotime) secondary antibodies, or co-incubated with Bodipy 20 *μ*g/ml and washed with PBS. Nuclei were stained by 4′,6-diamidino-2-phenylindole (DAPI; Beyotime) for 5 min. Aortic paraffin sections were prepared as previously described. Briefly, serial frozen sections (6 *μ*m) were pretreated in cold acetone for 10 min, washed with PBS, blocked for endogenous peroxidase activity, preincubated with goat serum, and then stained with anti-Lc3 (1 : 100, CST (Shanghai) Biological Reagents Company Limited) or anti-Moma-2 antibodies (1 : 100, AbD Serotec) for 1 h and washed with PBS, and then cells were incubated for 1 h with Alexa 488-conjugated (1 : 1000, Beyotime), Alexa 555-conjugated (1 : 500, Beyotime) secondary antibodies or co-incubated with Bodipy 20 *μ*g/ml and washed with PBS. Nuclei were stained by DAPI for 5 min. All slides were observed under a confocal laser microscopy (LSM780, Carl Zeiss, Oberkochen, Germany).

### Metabolic studies

TC, TGs, LDL and HDL levels were detected with an automated chemically modified technique (Roche Modular DPP System, Roche, Basel, Switzerland). For blood glucose measure, mice were fasted for 6 h, and the glucose concentration in whole blood collected from the tail vein was measured with a one touch Ultra In-Vitro Diagnosticum (Life Scan, Inc., Milpitas, CA, USA).

### PCR and real-time PCR

For determining whether the transplantation was successful, the genomic DNA was extracted from peripheral blood of ldlr^−/−^ mice after bone marrow transplantation and used as temple for PCR. PCR was performed using Pdcd4 or ldlr-specific primers as shown in Supplementary Table S1. For detection of gene expression on mRNA level, total RNA was extracted from cells using Trizol Reagent (Invitrogen). Real-time PCR was performed using UltraSYBR Mixture (CWBIO, Beijing, China) and specific primer pairs. The sequences of the sense and antisense primers were as shown in Supplementary Table S1. Data of relative molecule expression were presented by real-time quantitative PCR using the ΔΔCt model. Using the 2^ΔΔCt method, our data are reported as the fold change in experimental group normalized to an endogenous reference gene (18S) and relative to control group.

### Statistical analysis

All analyses were performed using the GraphPad Prism 5 program (GraphPad Software, Inc., San Diego, CA, USA). Unpaired Student's *t*-tests were used for the comparisons. Data are shown as mean ±S.E.M. A probability value of *P*<0.05 was considered significant.

## Abbreviations

PDCD4: programmed cell death 4
LD: lipid droplet
LDL: low-density lipoprotein
LC3: microtubule-associated protein 1 light chain 3
3-MA: 3-methyladenine

## Supplementary Material

Supplementary Informations
