# Supplementary material for: Pdcd4 deficiency enhances macrophage lipoautophagy and attenuates foam cell formation and atherosclerosis in mice
Source: Cell Death Dis. 2016 Jan 21;7(1):e2055–. doi: 10.1038/cddis.2015.416 (PMC4816189; doi:10.1038/cddis.2015.416)
Supplement: Supplementary Informations [file cddis2015416x1.doc]

**Pdcd4 deficiency enhances macrophage lipoautophagy and attenuates foam cell formation and atherosclerosis in mice**

**Supplemental Tables**

**Table S1: Primers used in PCR**

| **Primers** | **Sequences** |
| --- | --- |
| Abca1 | 5’-AGCCCGGAGATTCTTGGA-3’ |
|  | 5’-CACTGCCAAGGCACCTGAAC-3’ |
| Abcg1 | 5’-AGGGACACGATTCGCCTTT-3’ |
|  | 5’-GTCCACCCAACACCCATTCT-3’ |
| Lxr-α | 5’- CTCTGGAGGCTGCTGGGATTAG- 3’ |
|  | 5’- TTCCTGGAGCCCTGGACATTAC-3’; |
| 18S | 5’-AACCCGTTGAACCCCATT-3’ |
|  | 5’- CCATCCAATCGGTAGTAGCG-3’. |
| Pdcd4-wt | 5’-AGCCATTTCAGCCTTGGTGC-3’ |
|  | 5’-AATCTGTGTCTATGGTGAGGGTGG-3’ |
| Pdcd4-ko | 5’-GTTTGGAGGGAGGAAATGGAAG-3’ |
|  | 5’-AATCTGTGTCTATGGTGAGGGTGG-3 |
| Ldlr-wt | 5’-CCATAT GCATCCCCAGTCTT-3’ |
|  | 5’-GCGATGGATACACTCACTGC-3’ |
| Ldlr-ko | 5’-CCATATGCATCCCCAGTCTT-3’ |
|  | 5’-AATCCATCTTGTTCAATGGCCGATC-3’ |

**Table S2:** **The effect of Pdcd4 deficiency on body weight and metabolic parameters in high fat fed mice** **(16 weeks)**

|  | **apoe^-/-^(n=7)** | **DKO (n=5)** | **P value** |
| --- | --- | --- | --- |
| Body weight (g) | 30.48±1.05 | 28.84±0.86 | 0.11 |
| Blood glucose (mmol/L) | 8.44±0.51 | 10.30±0.65 | 0.06 |
| TCH (mmol/L) | 26.26±2.15 | 30.03 ±1.59 | 0.15 |
| TG (mmol/L) | 2.18±0.13 | 2.30±0.09 | 0.57 |
| HDL (mmol/L) | 8.77±0.49 | 8.01 ±0.33 | 0.63 |
| LDL (mmol/L) | 19.75±1.18 | 19.83 ±0.80 | 0.85 |

TC: Total plasma cholesterol; TG: triglycerides; HDL: high-density lipoprotein; LDL: low-density lipoprotein.

**Table S3:** **Change of body weight and metabolic parameters of ldlr^-/-^ mice after bone marrow transplantation**

|  | **WT→ldlr^-/-^ *(n=7)*** | **pdcd4^-/-^→ldlr^-/-^ *(n=7)*** | **P value** |
| --- | --- | --- | --- |
| Body weight (g) | 22.63±0.81 | 24.60±1.01 | 0.74 |
| TCH (mmol/L) | 19.18±2.32 | 17.75±0.51 | 0.61 |
| TG (mmol/L) | 4.09±0.85 | 2.81±0.05 | 0.13 |
| HDL (mmol/L) | 4.34±0.21 | 4.87±0.04 | 0.13 |
| LDL (mmol/L) | 11.02±0.38 | 11.07±0.51 | 0.94 |

TC: Total plasma cholesterol; TG: triglycerides; HDL: high-density lipoprotein; LDL: low-density lipoprotein.

**Supplemental Figures**


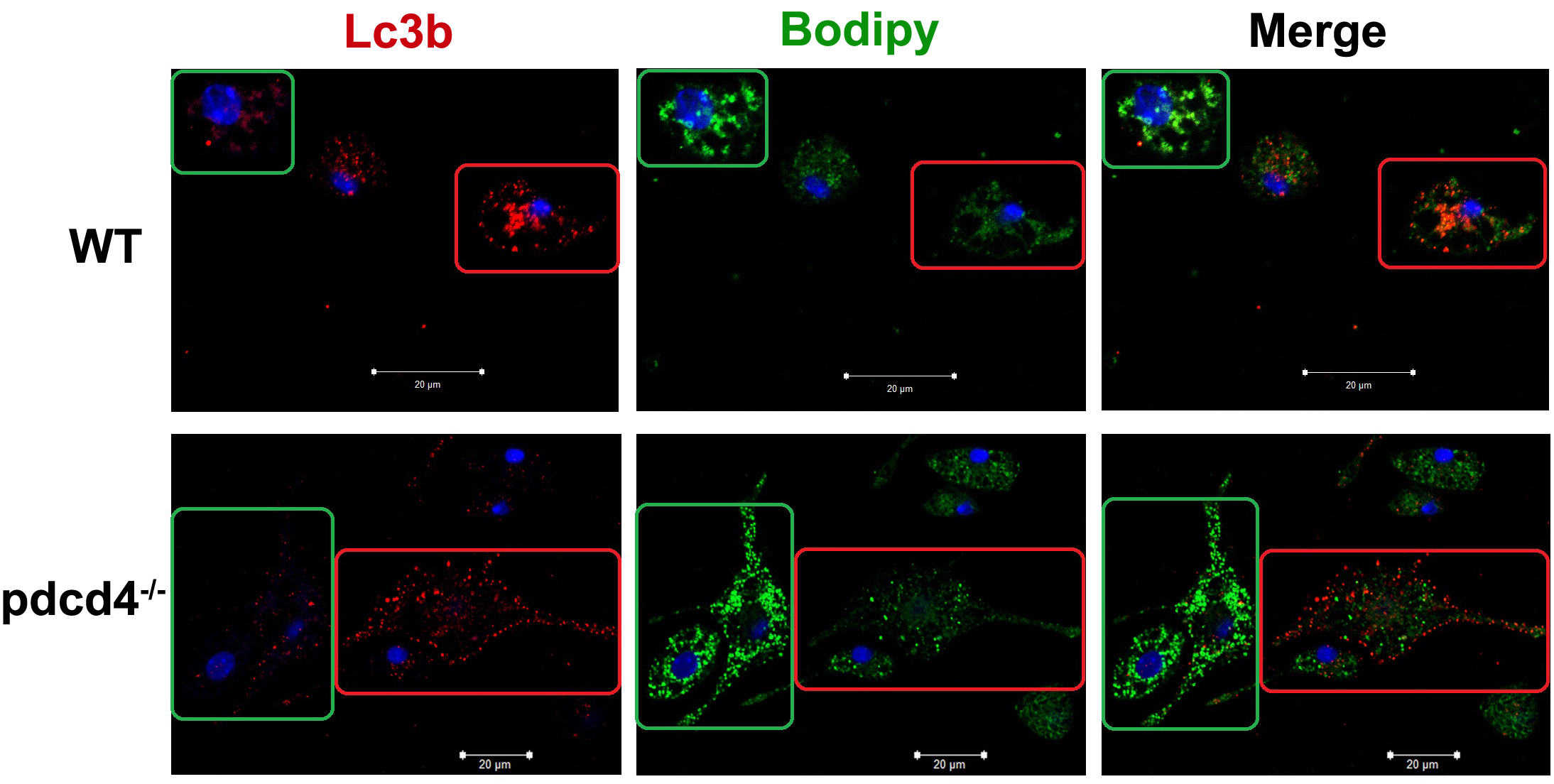


**Figure S1: Co-localization of lipid droplets with autophagosomes.** Peritoneal macrophages from wild type C57BL/6 and pdcd4^-/-^ mice were stimulated with 50 μg/mL of ox-LDL for 24h and then autophagosomes and lipid droplets were colocalized by double fluorescence staining with anti-Lc3b antibody (Red) and Bodipy (green).

**
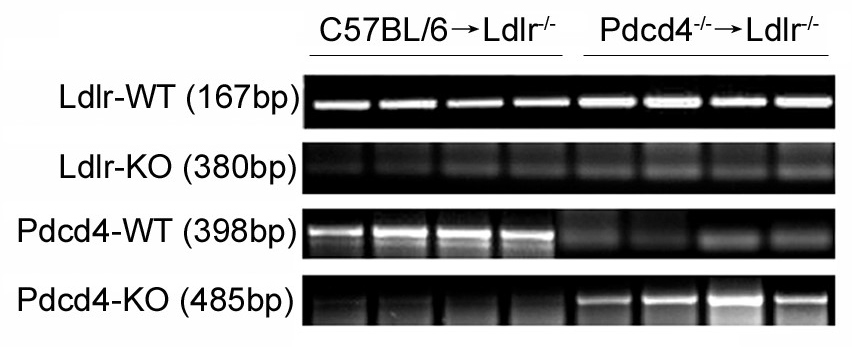
**

**Figure S2:** **Detection of ldlr^-/-^ mice gene type after the bone marrow transplantation by PCR.** The genomic DNAs were extracted from peripheral blood cells of ldlr^-/-^ mice after bone marrow transplantation and used as temple for PCR. The gene types were analyzed by wild type (Pdcd4-wt), pdcd4^-/-^ (Pdcd4-ko), wild type→ldlr^-/-^ (Ldlr-wt**)** and pdcd4^-/-^→ldlr^-/-^ (Ldlr^-/-^-ko) specific primers.
